# MouseMine: a new data warehouse for MGI

**DOI:** 10.1007/s00335-015-9573-z

**Published:** 2015-06-20

**Authors:** H. Motenko, S. B. Neuhauser, M. O’Keefe, J. E. Richardson

**Affiliations:** The Jackson Laboratory, Bar Harbor, ME 04609 USA; The MITRE Corporation, Boston, MA USA

## Abstract

MouseMine (www.mousemine.org) is a new data warehouse for accessing mouse data from Mouse Genome Informatics (MGI). Based on the InterMine software framework, MouseMine supports powerful query, reporting, and analysis capabilities, the ability to save and combine results from different queries, easy integration into larger workflows, and a comprehensive Web Services layer. Through MouseMine, users can access a significant portion of MGI data in new and useful ways. Importantly, MouseMine is also a member of a growing community of online data resources based on InterMine, including those established by other model organism databases. Adopting common interfaces and collaborating on data representation standards are critical to fostering cross-species data analysis. This paper presents a general introduction to MouseMine, presents examples of its use, and discusses the potential for further integration into the MGI interface.

## Introduction

The Mouse Genome Informatics consortium (MGI) has a long history of delivering comprehensive, high-quality online information about the genetics, genomics, and biology of the laboratory mouse (Eppig et al. 2015; Bult et al. 2015; Smith et al. 2014). To maximize the use of these data, MGI has always provided multiple means to access the information. The main web interface (www.informatics.jax.org) supports interactive database querying, viewing, and downloading. A “Batch Query” tool (www.informatics.jax.org/batch) supports uploading a list of gene IDs/symbols and getting back certain information about those genes. Two MGI BioMart databases (available at biomart.informatics.jax.org) support access to basic information about mouse genes (IDs, symbols, alleles, coordinates, GO and MP terms, and orthologs) and to gene expression annotations. Finally, MGI provides many database reports and a public read-only copy of the database to support direct SQL querying (contact: mgi-help@jax.org).

MouseMine (www.mousemine.org) is the latest step in the evolution of MGI online services. Based on the InterMine (Smith et al. 2012; www.intermine.org) data warehouse system, MouseMine supports powerful query, reporting, and analysis capabilities over a significant portion of the MGI database. MouseMine is a member of a growing community of mines and in particular, is a member of InterMOD (Sullivan et al. 2013), a consortium of mines developed by several model organism databases (MODs). The combination of Intermine’s capabilities and its growing adoption among the MODs significantly enhances a user’s ability to do cross-species data mining and analysis.

## InterMine and InterMOD

InterMine is an open source software framework originally developed to support a *Drosophila* data warehouse called FlyMine (Lyne et al. 2007). Following this initial success, a new project then generalized the software, calling it InterMine (Smith et al. 2012), and established mines for three other model organisms: rat (RGD/RatMine), yeast (SGD/YeastMine), and zebrafish (ZFIN/ZebrafishMine). Starting in 2012 mines were established for mouse (MGI/MouseMine) and worm (WormBase/WormMine). This consortium of MODs, called InterMOD, works together and with the InterMine team, communicating regularly on common data issues, representation standards, and interfaces. Mines for additional species (e.g., human, *Xenopus*, and *Arabidopsis*) are also being established though other funding. Users can now access data for multiple species using common interfaces and tools, e.g., a user familiar with FlyMine can immediately start using MouseMine.

**Fig. 1:**
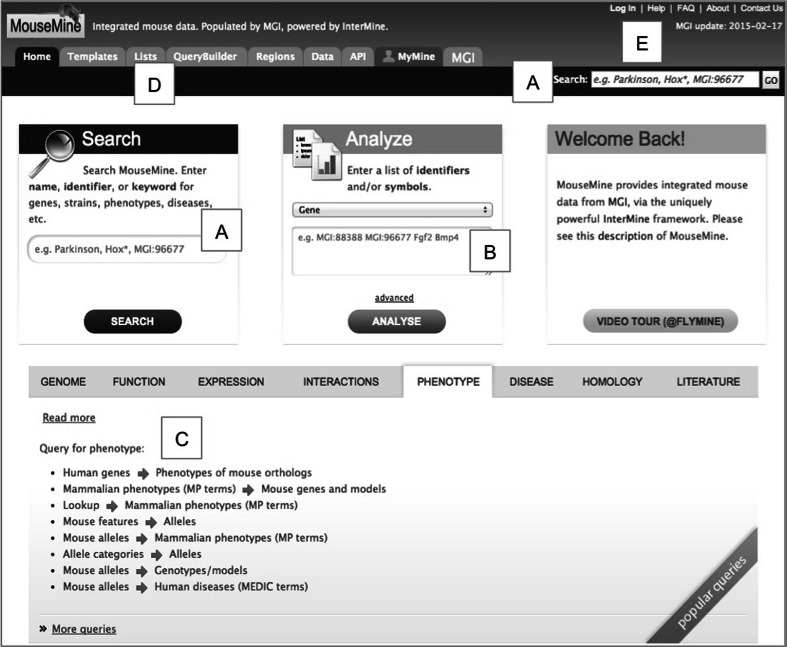
MouseMine home page (www.mousemine.org). The user can perform a keyword search (*a*), upload gene, or other IDs to create a list (*b*), or run one of the available queries (*c*). Other tabs (*d*) provide additional features such as the ability to create/edit custom queries or perform a region search. Users may optionally log in (*e*) in order to save lists and custom queries permanently. Other features on the home page include links to help, contact information, and the date of the most recent update from MGI

**Fig. 2:**
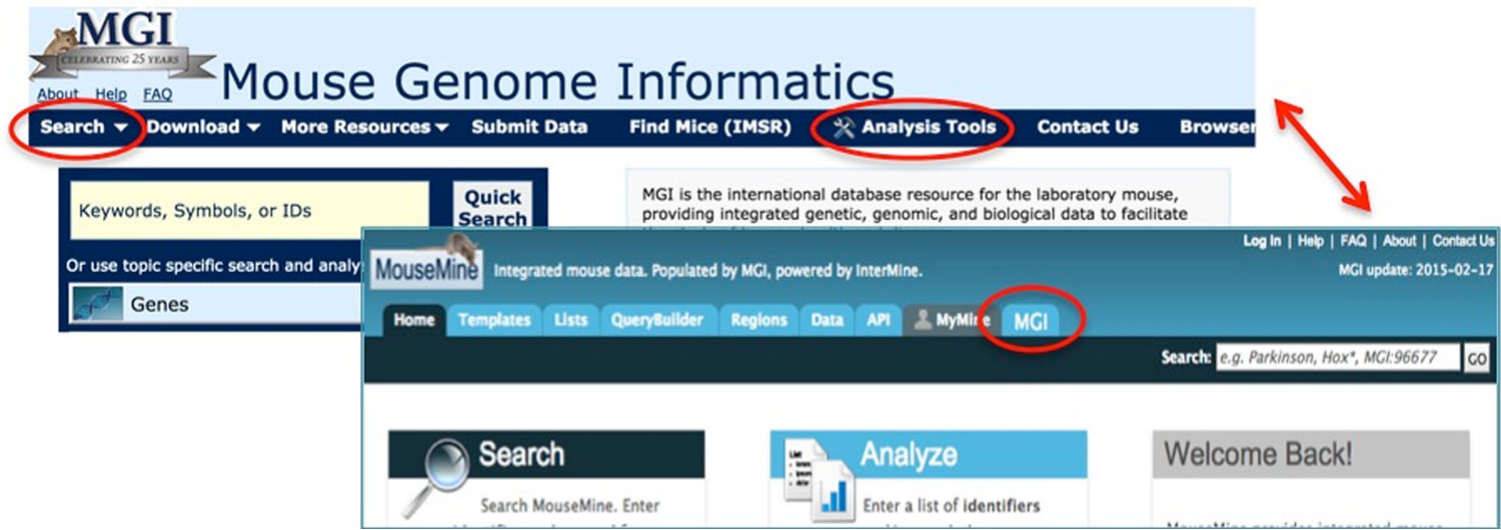
Top level links. Links to MouseMine from MGI are found on the main search menu and on the analysis tools page. A link to MGI from MouseMine is at the top of every MouseMine page

As part of the InterMOD project, MouseMine was established as a new vehicle for delivering MGI data and for collaborating with the other MODs. The MouseMine home page is shown in Fig. 1, while the main links for navigating between MGI and MouseMine are shown in Fig. 2. MouseMine contains core curated data from MGI, including the mouse genome feature catalog with nomenclature, genome coordinates, and database cross-references; the catalog of engineered and spontaneous alleles; mouse strains and genotypes; ontologies and annotations, including function (GO-to-feature), phenotype (MP-to-genotype), and disease (OMIM-to-genotype); gene expression data and developmental mouse anatomy; orthology and paralogy data from Homologene and Panther; curator notes; and publications.

## Features

**Fig. 3:**
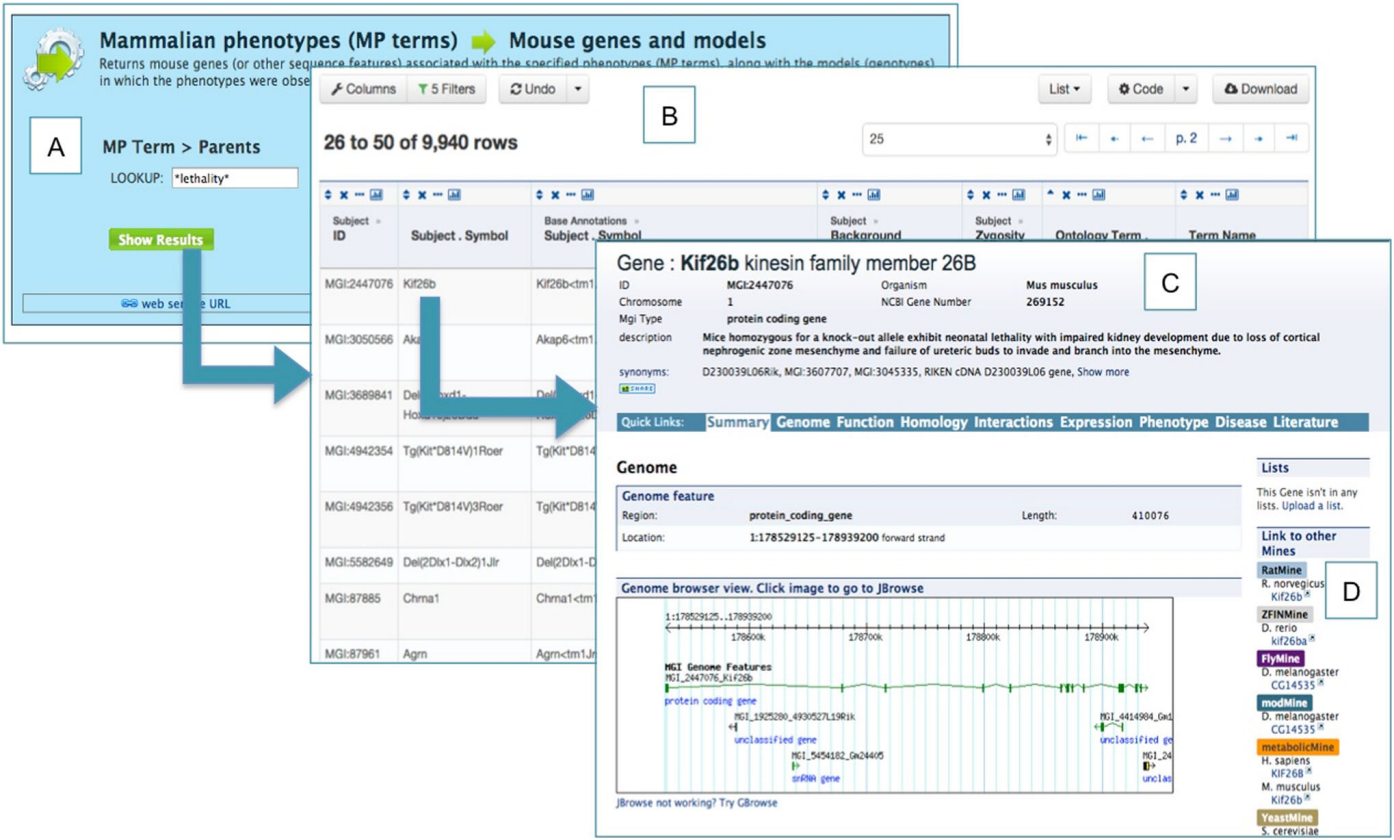
MouseMine offers query forms (*a*), tables of results (*b*), and detail pages for genes and other objects (*c*). Query forms tend to be very simple because results tables are so flexible, allowing easy filtering and customization after the initial query. Detail pages offer a variety of displays, e.g., graphical maps and result tables of data specific for that object. Gene pages in the different mines link to one another automatically, based on orthology relations (*d*)

MouseMine provides many powerful features that are included with InterMine “out of the box” and which we have customized for our installation. For users, there is a complete web interface with familiar features such as faceted keyword searching, detail pages for individual objects (e.g., genes), query forms (called “templates”), and result tables. A flexible table display is used throughout the interface that provides many features for interacting with query results. This includes paging, sorting (including multilevel sorts), filtering, removing/adding/reordering columns, and downloading in multiple formats. Many predefined templates provide a variety of starting points for exploration. Figure 3 shows one example. For users who wish to go beyond the provided query forms, there is a point-and-click interface for building and customizing ones own template queries. MouseMine supports both anonymous public use and user logins; a logged-in user can save custom queries and lists (lists are discussed below) permanently, while an anonymous user’s lists and queries only last the session. For computational users, InterMine provides a comprehensive web service API (Kalderimis et al. 2014) that supports running any query, performing keyword searches, creating and manipulating lists, indeed, accessing any of the functionality available through the user interface. Programmers may access the RESTful end points directly or use a client library in one of several languages. For mine developers, InterMine provides an extensible object-oriented type system that facilitates modeling the data one intends to load, many ready-to-use components for loading common datasets (e.g., Panther, BioGrid, Entrez, etc.), the ability to write custom loaders, a query engine (built on PostgreSQL) that uses pre-computation and results caching to achieve high performance.

## List management

**Fig. 4:**
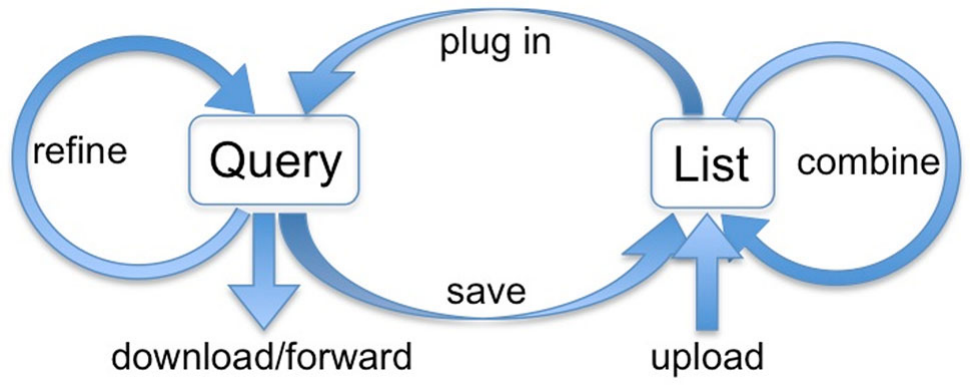
InterMine allows iterative workflows. From any query result, the user may further refine those results and then save a list of objects, e.g., genes, publications, GO terms, etc. In turn, saved lists can be combined (using standard set operations), and a list can be “plugged” into a query, running the query over that specific set of objects

**Fig. 5:**
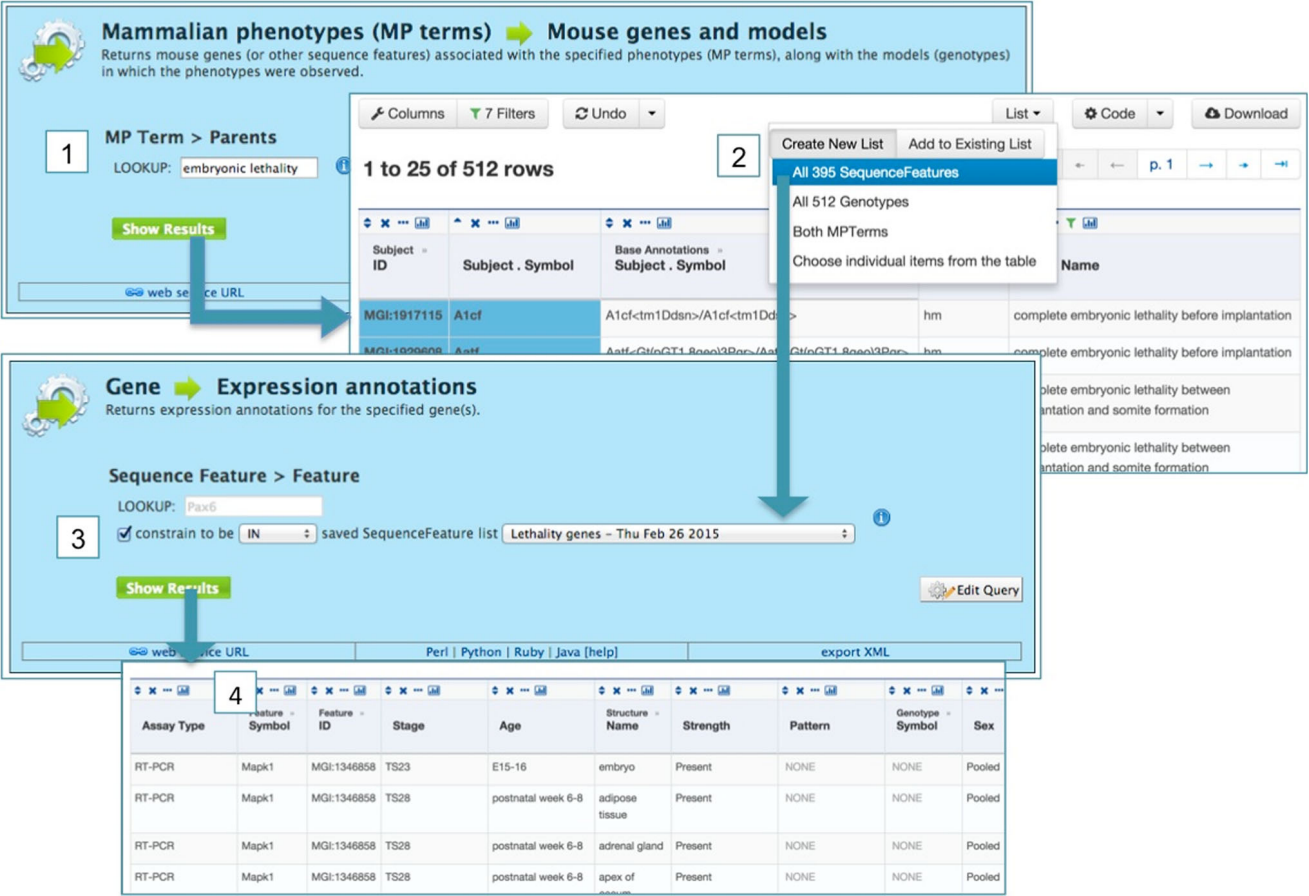
The ability to save lists from queries, to combine saved lists in various ways, and to “plug” those lists into other queries, all combine to give the user great power and flexibility. Complex tasks can be broken down into a series of simple steps. In this example, the user wants the expression data for genes associated with certain embryonic lethality phenotypes in homozygous mutants. In the first panel (*1*), the user queries for all mouse genes and models (genotypes) associated with embryonic lethality. In the next panel (*2*), the user has filtered the results down to homozygous mutants and the particular forms of lethality (steps not shown) and is now saving the list of 395 remaining genes. In the next panel (*3*), the user “plugs” the new list, named “Lethality genes” (naming step not shown), into a query that returns all the expression data for those genes (*4*)

A key InterMine user feature, and a significant enhancement for MGI users, is the ability to create and manage lists of objects, e.g., lists of genes, GO terms, publications, etc., and to use those lists to drive further queries. These two capabilities combine to enable powerful iterative querying and workflows, as illustrated in Fig. 4. A user can create a list by uploading a set of IDs (e.g., Ensemble IDs for genes or PubMed IDs for publications) or by saving results from a query. Lists can be combined using set operators (union, intersection, difference). A list can also “drive” a query in that many template queries will accept a list of objects as input and will run against that list. For example, one template returns the GO annotations for a gene entered by a user. The same template will also return GO annotations for all the genes in a specified list. An example of this is shown in Fig. 5.

## Data sources

The main source of MouseMine data is MGI, which includes a wealth of information about the structure and function of the mouse genome, developmental gene expression patterns, phenotypic effects of mutations, and annotations of human disease models. These data also include a rich set of cross-references (e.g., EntrezGene, UniProt, OMIM, etc.) and cross-species associations (e.g., orthologies to human, rat, zebrafish, etc.), allowing the user to make critical connections to other data resources.

The main software development component in building MouseMine is the code to extract the data from MGI, restructure it to match the InterMine data model (or sometimes, extend the model to match the MGI data), and output it as a set of XML files in a specific format defined by InterMine. This component, called “the dumper”, is also the main source of maintenance costs for MouseMine, as it needs to keep up with the regular changes in MGI. Fortunately, the InterMine data model is both remarkably close to MGI’s in essential ways and is easily extended when needed. This allows the restructuring parts of the dumper to be relatively straightforward and is a significant technical advantage of InterMine over BioMart.

MouseMine also loads data from several other sources in addition to MGI. In most cases, we exploit source loaders already included with InterMine. For example, the NCBI Taxonomy database supplies basic nomenclature information for organisms, and ontologies are loaded from OBO files downloaded from several sources (e.g., the OboFoundry). A more interesting example is Publications. Most InterMine data loaders only create publication “stubs”, i.e., objects having only a PubMed id. InterMine supplies a loader, usually one of the last to run when building a mine, which accesses PubMed and fills in all the details (title, authors, journal, date, etc.) for every publication with a PMID. (Details for the handful of publications without PMIDs come from MGI.) MouseMine also contains a small but growing segment of data not found in MGI such as interactions from BioGrid and IntAct, and homology data from Panther.

## Build infrastructure

MouseMine is rebuilt each week (or whenever there is a data refresh at MGI). The MouseMine build process is completely automated and is controlled by Jenkins, a widely used job management system. A build proceeds in several phases. The first phase prepares all the data files needed to load MouseMine (including running the MGI dumper), the second phase loads/integrates those files into the mine, and a third runs a series of acceptance tests to ensure that the result is consistent with MGI. If (and only if) all tests pass, the results are then “pushed out” to the publicly accessible server.

**Fig. 6:**
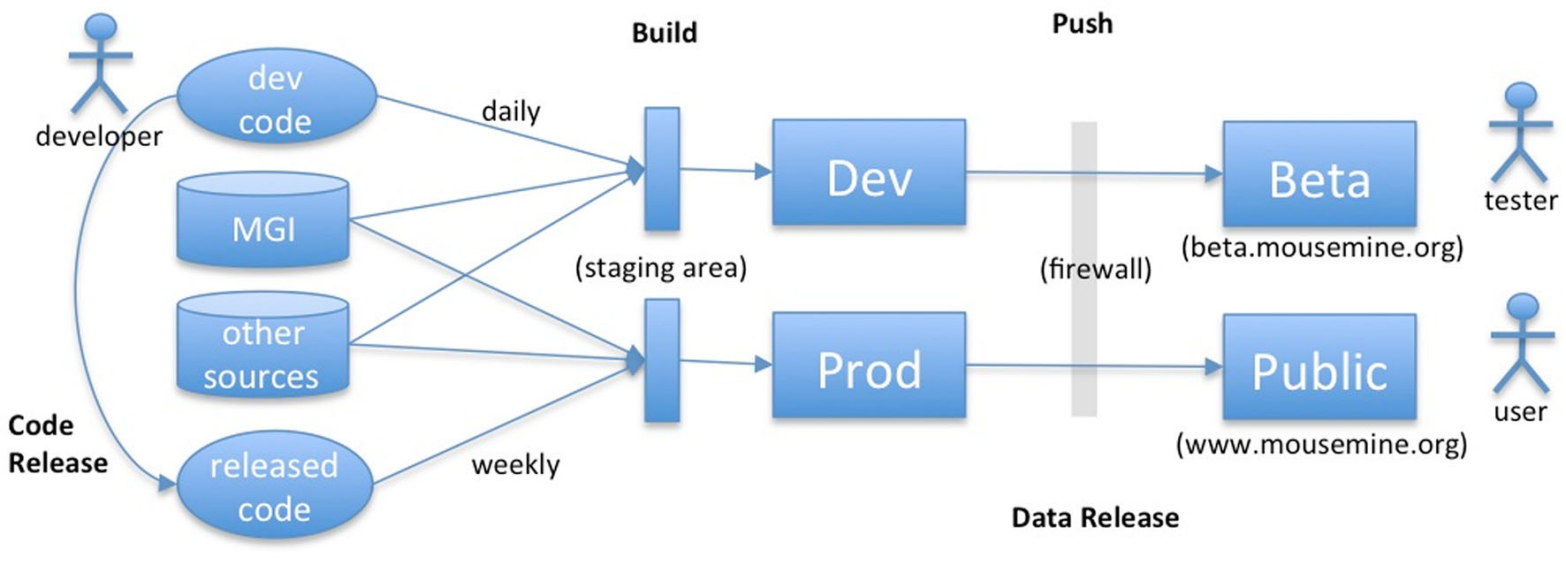
MouseMine build process. MouseMine builds are completely automated. The build process pulls data from MGI and a few other sources (e.g., ontologies are loaded from the OBO Foundry) to a staging area for loading/integrating in the mine. Each build is verified by a series of acceptance tests to ensure consistency with MGI. A successful build is then “pushed” across the Lab’s secure firewall to a public server. New development and testing, e.g., importing additional data types or developing new templates, happen on a Dev server and are pushed to a publicly accessible Beta server for preview. Sets of completed changes are then grouped and tagged for release and are included in the next build on Prod/Public

MouseMine is supported by five virtual Linux servers running on a pair of hardware servers (blades) in a local cloud at The Jackson Laboratory. One virtual server (dev) supports new software development (e.g., loading additional data types) and daily builds. A beta server supports pre-release access to new features and new datasets for vetting and verification. Another server (prod) runs the weekly build for the public update, while the public website (pub) runs on a fourth server. The fifth server (test) supports updating third party components such as InterMine or Postgres with new versions. Because both pub and beta support public access, they exist outside The Jackson Laboratory firewall; the development, test, and build servers are inside. New builds for public and for beta run on internal servers (prod and dev, respectively) and are then pushed to the public side. Figure 6 shows a high-level view of this process.

## Discussion

Agencies that fund MODs are concerned with their long-term sustainability. While this is a complicated issue with no “magic” solutions, the adoption of InterMine by the MODs is a step in the right direction. The tool is powerful, flexible, and free; a mine can be established and maintained with relatively modest effort; users can, for the first time, access all the MODs using a common interface; and contributions to the tool made by one benefit all.

For MGI, MouseMine represents the latest step in its ongoing efforts to disseminate high-quality comprehensive mouse data to the widest audience, to provide powerful programmatic access, to cooperate with other MODs to foster cross-species data analysis, and to embrace strategically important new technologies. Plans for MouseMine include loading additional MGI data, such as miRNA-target interactions and gene models, as well as data from other MGI resources such as cancer models from the Mouse Tumor Database and metabolic pathway data from MouseCyc.

While MouseMine provides a complete web interface, it is also possible to take components of that interface and embed them in other web pages. In particular, it is easy to embed a table showing the results of any desired query and providing all of the interactive functionality available through MouseMine. We can use this capability to augment current MGI web pages. For example, MGI currently provides a page that displays all the phenotype annotations for one or more genotypes, formatted for reading. With relative ease, we could augment this page with the option to see the underlying annotation records, with the ability to sort/filter/download/etc. We can also leverage this functionality to embed other visual components included with InterMine, such as a map displays, protein interaction displays, and a generic graph widget. And finally, the comprehensive web services API provides an open-ended interface for building new interactive and embeddable displays.
